# Alteration and clinical potential in gut microbiota in patients with cerebral small vessel disease

**DOI:** 10.3389/fcimb.2023.1231541

**Published:** 2023-07-11

**Authors:** Yachen Shi, En Zhao, Lei Li, Songyun Zhao, Haixia Mao, Jingyu Deng, Wei Ji, Yang Li, Qianqian Gao, Siyuan Zeng, Lin Ma, Guangjun Xi, Yiping You, Junfei Shao, Xiangming Fang, Feng Wang

**Affiliations:** ^1^ Department of Neurology, the Affiliated Wuxi People’s Hospital of Nanjing Medical University, Wuxi People’s Hospital, Wuxi Medical Center, Nanjing Medical University, Wuxi, China; ^2^ Department of Interventional Neurology, the Affiliated Wuxi People’s Hospital of Nanjing Medical University, Wuxi People’s Hospital, Wuxi Medical Center, Nanjing Medical University, Wuxi, China; ^3^ Department of Gastroenterology, Xishan People’s Hospital of Wuxi City, Wuxi, China; ^4^ Department of Neurosurgery, the Affiliated Wuxi People’s Hospital of Nanjing Medical University, Wuxi People’s Hospital, Wuxi Medical Center, Nanjing Medical University, Wuxi, China; ^5^ Department of Radiology, the Affiliated Wuxi People’s Hospital of Nanjing Medical University, Wuxi People’s Hospital, Wuxi Medical Center, Nanjing Medical University, Wuxi, China; ^6^ Department of Functional Neurology, the Affiliated Wuxi People’s Hospital of Nanjing Medical University, Wuxi People’s Hospital, Wuxi Medical Center, Nanjing Medical University, Wuxi, China

**Keywords:** cerebral small vessel disease, gut microbiota, cognitive function, magnetic resonance imaging, least absolute shrinkage and selection operator algorithm

## Abstract

**Background:**

Cerebral small vessel disease (CSVD) is a cluster of microvascular disorders with unclear pathological mechanisms. The microbiota-gut-brain axis is an essential regulatory mechanism between gut microbes and their host. Therefore, the compositional and functional gut microbiota alterations lead to cerebrovascular disease pathogenesis. The current study aims to determine the alteration and clinical value of the gut microbiota in CSVD patients.

**Methods:**

Sixty-four CSVD patients and 18 matched healthy controls (HCs) were included in our study. All the participants underwent neuropsychological tests, and the multi-modal magnetic resonance imaging depicted the changes in brain structure and function. Plasma samples were collected, and the fecal samples were analyzed with 16S rRNA gene sequencing.

**Results:**

Based on the alpha diversity analysis, the CSVD group had significantly decreased Shannon and enhanced Simpson compared to the HC group. At the genus level, there was a significant increase in the relative abundances of Parasutterella, Anaeroglobus, Megasphaera, Akkermansia, Collinsella, and Veillonella in the CSVD group. Moreover, these genera with significant differences in CSVD patients revealed significant correlations with cognitive assessments, plasma levels of the blood-brain barrier-/inflammation-related indexes, and structural/functional magnetic resonance imaging changes. Functional prediction demonstrated that lipoic acid metabolism was significantly higher in CSVD patients than HCs. Additionally, a composite biomarker depending on six gut microbiota at the genus level displayed an area under the curve of 0.834 to distinguish CSVD patients from HCs using the least absolute shrinkage and selection operator (LASSO) algorithm.

**Conclusion:**

The evident changes in gut microbiota composition in CSVD patients were correlated with clinical features and pathological changes of CSVD. Combining these gut microbiota using the LASSO algorithm helped identify CSVD accurately.

## Introduction

Cerebral small vessel disease (CSVD) is a prevalent neuropathological process in clinical practice and is vital in dementia, stroke, depression, and gait disturbances among elderly patients (Cannistraro et al., 2019; Markus and Erik De Leeuw, 2023). Disrupting the blood-brain barrier (BBB), neuroinflammation, and impaired neurovascular coupling can cause CSVD (Ungvari et al., 2021). However, the underlying pathogenesis remains poorly understood. Currently, the clinical diagnosis of CSVD is based on brain magnetic resonance imaging (MRI) features, such as recent small subcortical infarcts, white matter hyperintensities (WMH), lacunar infarcts (Lis), cerebral microbleeds (CMBs), and enlarged perivascular spaces (EPVS) (Chen et al., 2019b; Van Den Brink et al., 2023). Additionally, various circulating blood indexes are considered potential biomarkers to identify and reflect the pathological changes in CSVD. These include matrix metalloproteinase-9 (MMP-9) in BBB integrality (Li et al., 2022), neurofilament light in axonal injury (Qu et al., 2021), and tumor necrosis factor-alpha (TNF-α) in inflammatory response (Wan et al., 2023). Meanwhile, advanced imaging also provides new insight into CSVD pathophysiology. Resting-state functional MRI depicts the association between altered brain function in the sensorimotor and frontoparietal networks and gait disorders (Zhou et al., 2020) and the underlying mechanism of aberrant spontaneous brain activity within the default mode network, leading to cognitive decline in CSVD (Li et al., 2021).

Gut microbes can develop neuroactive compounds and regulate neuronal function, vital in gut-brain interactions (Ascher and Reinhardt, 2018; Pluta et al., 2021). The gut-brain axis is necessary for the onset and progression of cerebrovascular disease through complex signaling pathways. These involve vagus nerves within the enteric nervous system, the neuronal-glial-endothelial interactions, and activating gut inflammatory and immune cells induced by cytokines (Arya and Hu, 2018). The microbial-derived metabolites (*e.g.*, short-chain fatty acids, trimethylamines, amino acid metabolites) play an essential role in metabolic and signaling functions of BBB and brain neurons to protect from the pathology and inflammation asstociated with disease (Parker et al., 2020; Hoyles et al., 2021). However, the intestinal ecological imbalance can affect the global immune system and alter the production of neuroprotective intestinal metabolites (Huang and Xia, 2021; Nelson et al., 2021). This causes aggravation of neuroinflammation and BBB dysfunction. Previous studies have demonstrated that stroke patients have reduced gut microbiome diversity, and several microbial taxa, such as *Streptococcus*, *Lactobacillus*, *Escherichia*, *Eubacterium*, and *Roseburia*, could be risk indicators of ischemic stroke (Peh et al., 2022; Zou et al., 2022). Cai et al. recently observed that gut microbiota could up-regulate interleukin-17A production in neutrophils by activating RORγt signaling to induce CSVD occurrence (Cai et al., 2021). Cerebral autosomal dominant arteriopathy with subcortical infarcts and leukoencephalopathy also depicts significant changes in gut microbiota abundance at the genus level, and this could lead to the onset and progression of hereditary CSVD (Matsuura et al., 2019). Since these findings are in the initial stage, the underlying association of gut microbiota with CSVD should be investigated. Therefore, recognizing the implication of the gut microbiota in CSVD pathophysiology and identifying beneficial gut bacteria are crucial as early warning CSVD biomarkers.

The current study aimed to determine the gut microbiota alteration and underlying pathological mechanisms in CSVD patients. Additionally, the relationship between the clinical features of CSVD and the expression of the gut microbiota was thoroughly investigated. Meanwhile, machine learning helped build a composite biomarker depending on the gut microbiota in CSVD. The current study also assessed the diagnostic potential of the gut microbiota to identify CSVD early.

## Materials and methods

### Participants

64 CSVD patients were recruited from the Affiliated Wuxi People’s Hospital of Nanjing Medical University, and 18 matched healthy controls (HCs) were included in the present study through community health screening. All participants were Chinese Han. Neuropsychological assessments including Mini-Mental State Examination (MMSE), and Montreal Cognitive Assessment (MoCA), and 17-Item Hamilton Depression Rating Scale (HAMD-17), were carried out on all the recruited participants. Moreover, National Institutes of Health Stroke Scale (NIHSS) was also used for evaluate the symptom of stroke. Furthermore, the multi-modal MRI including the structural MRI and functional MRI, were conducted in all the participants.

All the participants provided a written informed consent. The Ethics Committee of the Affiliated Wuxi People’s Hospital of Nanjing Medical University approved the present study (approval number: KY2112).

### Inclusion and exclusion criteria

The inclusion criteria for all the participants were: (1) aged 50-80 years old; (2) ≥ six years of education; and (3) no contraindication in MRI scan.

CSVD patients were diagnosed according to the established diagnostic criteria (Chen et al., 2019b) using MRI evidence of vascular changes. Participants were found as the CSVD patients with the following features: (1) total number of lacunar infarcts (Lis) were counted, and ≥ 2 Lis were considered as the presence of lacunes; (2) periventricular and deep WMH was quantified using the Fazekas scale (overall score of 3), and a score ≥ 1 was considered as displaying WMH; (3) the total number of CMBs were counted, and ≥ 1 CMBs was considered as a positive; or (4) the total number of EPVS were counted, and ≥ 10 EPVS was considered as a threshold. Additionally, all the matched HCs had not any stroke performance, as reflected by no imaging changes (**Table 1**).

**Table 1:** Comparison of demographic and clinical characteristics of subjects between the CSVD and HC groups.

	CSVD (n = 64)	HC (n = 18)	*P-value*
Age (years)	69.15 ± 5.73	66.17 ± 5.49	0.052^*^
Sex (male/female)	32/32	7/11	0.437^&^
Education years	8.83 ± 2.43	8.89 ± 2.42	0.782^#^
Hypertension (yes/no)	38/26	9/9	0.592^&^
Diabetes (yes/no)	16/48	2/16	0.335^&^
NIHSS scores	0.48 ± 0.73	0	–
MMSE scores	27.59 ± 2.20	28.67 ± 1.24	0.032^#^
MoCA scores	24.52 ± 4.27	27.00 ± 2.09	0.023^#^
HAMD-17 scores	3.48 ± 3.98	2.33 ± 3.24	0.214^#^
Plasma S100β (pg/ml)	96.25 ± 26.03	71.07 ± 27.03	0.001^*^
Plasma MMP-9 (ng/ml)	25.42 ± 12.04	21.41 ± 5.48	0.538^#^
Plasma NSE (ng/ml)	49.85 ± 22.41	13.70 ± 9.16	< 0.001^*^
Plasma TNF-α (pg/ml)	8.15 ± 2.07	6.20 ± 1.64	< 0.001^*^
Structural MRI features			
Periventricular WMH Fazekas scores	1.80 ± 0.67	0	–
Deep WMH Fazekas scores	0.89 ± 0.89	0	–
Total WMH Fazekas scores	2.69 ± 1.38	0	–
Number of EPVS	4.45 ± 5.37	0	–
Number of CMBs	2.84 ± 3.53	0	–
Number of Lis	10.41 ± 8.49	0	–

CSVD, cerebral small vessel disease; HC, healthy control; NIHSS, National Institutes of Health Stroke Scale; MMSE, Mini-mental State Examination; MoCA, Montreal Cognitive Assessment; HAMD-17, 17-Item Hamilton Depression Rating Scale; S100β, S100beta protein; MMP-9, matrix metalloproteinase-9; NSE, neuron-specific enolase; TNF-α, tumor necrosis factor-alpha; MRI, magnetic resonance imaging; WMH, white matter hyperintensities; EPVS, enlarged perivascular spaces; CMBs, cerebral microbleeds; Lis, lacunar infarcts.

^*^P-values were obtained by Independent-Samples T test.

^#^P-values were obtained by Mann-Whitney U test.

^&^P-values were obtained by Chi-square test.

“-” means no statistic analysis was performed.

The exclusion criteria for each participant were: (1) clinical evidence supporting cerebrovascular disorders with large intracranial vascular lesions; (2) any severe psychiatric disorders (*e.g.*, schizophrenia); (3) abuse or alcohol or drugs dependence; (4) brain trauma or other neurologic diseases (*e.g.*, Parkinson’s disease); and (5) any significant medical problems (*e.g.*, tumor, significantly impaired liver or kidney functions).

### Functional MRI data acquisition and preprocessing

The imaging data preprocessing was performed using the Data Processing Assistant for Resting-State functional MRI (DPARSFA 2.3) toolbox (Chao-Gan and Yu-Feng, 2010). The amplitude of low-frequency fluctuation (ALFF) estimates the local spontaneous neuronal activity (Chen et al., 2022; Shi et al., 2023a). The REST software was used to calculate ALFF values and help perform the statistical analysis between the two groups (Alphasim multiple comparison correction p < 0.05). The brain regions with significant ALFF values were displayed using the BrainNet Viewer software. More details were displayed in **Supplementary Materials** and could be found in our previous studies (Shi et al., 2021a; Shi et al., 2021b; Shi et al., 2022).

### Collection of fecal samples and 16S rRNA gene sequencing

After overnight fasting, the fecal sample was collected (7:00-9:00 AM) after participant’s defecation using stool collection tubes with stool DNA stabilizer (Genstone Biotech, Beijing, China), and then stored at - 80°C.

The DNA extractions of fecal samples using FastDNA Spin Kit For Soil (MP Biomedicals, Santa Ana, CA) and compositional analysis of gut microbiota were performed by Genesky Biotechnologies Inc. (Shanghai, China). Details of sequencing and data analysis were provided in **Supplementary Materials** and previous study (Xu et al., 2022).

### Collection of plasma samples and detection of plasma indexes

Peripheral venous blood was collected using EDTA-coated tubes after the collection of fecal samples immediately. Then, the plasma samples were obtained by centrifugation at 2000 × g at 4°C for 10 minutes, and further were stored at -80°C until use.

Four plasma indexes, S100beta protein (S100β) (Cao et al., 2022), MMP-9 (Rempe et al., 2016), neuron-specific enolase (NSE) (Yun et al., 2022), and TNF-α (Zelová and Hošek, 2013), were detected using commercial enzyme-linked immunosorbent assays kit (FineTest, Wuhan, China; Catalog Number: EH0543 for S100β, EH0238 for MMP-9, EH0370-HS for NSE, and EH0302 for TNF-α). The levels of these indexes were measured in triplicate and the inter- and intra-assay coefficients of variation were < 5%.

### Statistical analyses

The data analyses were conducted using SPSS version 22.0 (SPSS Inc. Chicago, IL, USA) and R software package (version 4.2.1).

The diversity analysis of gut microbiota was comprised of alpha and beta diversity analyses. The alpha-diversity included Observed species, Chao1, and ACE indicators for the community richness and Shannon, Simpson, and Coverage for the community diversity. Beta diversity is used to indicate differences in the composition of gut microbiota using Partial Least Squares-Discriminant Analysis. Linear Discriminant Analysis (LDA) Effect Size (LEfSe) was conducted to identify the markers to interpret the difference between groups where the threshold score of LDA was two. The functions of species in the gut microbiota of both the groups were predicted using PICRUSt2 analysis tool and Kyoto Encyclopedia of Genes and Genomes database (https://www.genome.jp/kegg/pathway.html).

The Kolmogorov-Smirnov test was used to evaluate the normal distribution of the data. The continuous variables were shown as mean ± standard deviation and were analyzed using the Mann-Whitney U test for non-normal distribution, or the Student’s t-test for normal distribution. The categorical variables were analyzed using the chi-square test. Pearson correlation analysis was used to determine the correlation between the taxonomies of gut microbiota and clinical data and MRI data. Furthermore, to identify the potential diagnostic biomarker of gut microbiota for CSVD, least absolute shrinkage and selection operator (LASSO) algorithm was used to construct a composite biomarker based on the gut microbiota with significant difference between the groups (Ji et al., 2021; Shi et al., 2023b). Receiver operating characteristic (ROC) curves were utilized to compute the area under the curve (AUC) for determining the diagnostic accuracy of the biomarkers. The Youden index was used to estimate optimal values of sensitivity and specificity. The statistically significant differences were considered as P-value < 0.05.

## Results

### Characteristics of participants

**Table 1** represents no difference in age, sex, education years and complications (hypertension and diabetes) between the CSVD and HC groups. CSVD patients had significantly increased NIHSS, MMSE, and MoCA scores when compared to HCs. However, there was no significant difference in HAMD-17 scores between the two groups.

Additionally, the plasma levels of S100β, NSE, and TNF-α were significant higher in CSVD patients than HCs, however, plasma MMP-9 exhibited a similar level between the two groups (**Table 1**).

### Compositional analysis of gut microbiota in CSVD and HC groups

A total 5,357,333 sequences were obtained from 82 samples, including 4,160,196 sequences in the CSVD group, and 1,197,137 sequences in the HC group, after the quality filtration, noise reduction, splicing, and de-chimerism of data using QIIME2 software (SRA accession number: PRJNA985039).

The operational taxonomic units were assigned with a 95% sequence similarity threshold. The CSVD group exhibited a higher number of operational taxonomic units than the HC group (3463 vs. 1560), including 901 similar operational taxonomic units (**Supplementary Figure 1A**). All the samples’ curves in the rarefaction curves based on the amplicon sequence variant reached saturation plateau at the depth of 52,000 reads, indicating that the sequencing depths were sufficient to represent the majority of microbe species, and the number of samples was reasonable (**Supplementary Figure 1B**).

### Diversity analysis of gut microbiota between two groups

#### Alpha diversity analysis

Six α-diversity indicators, *i.e.*, Observed species, Chao1, ACE, Shannon, Simpson, and Coverage, were analyzed in the present study (**Supplementary Figure 2A**). Except for Coverage scores, significant reduced Shannon scores and significant elevated Simpson scores were found in the CSVD group when compared to the HC group, suggesting lower species diversity in the gut microbiota of the CSVD group. Meanwhile, compared with the HC group, Observed, Chao 1, and ACE scores displayed reduced trends in the CSVD group, which suggested lower species richness in the gut microbiota of the CSVD group.

#### Beta diversity analysis

The β-diversity was calculated using Partial Least Squares-Discriminant Analysis, a supervised discriminant analysis, for the reduction of the impact of intergroup differences (**Supplementary Figure 2B**). The results of β-diversity suggested that the overall composition of gut microbiota in the CSVD group patients was different from that of the HC group.

#### Compositional analysis of gut microbiota at genus level levels between two groups

**Figure 1:**
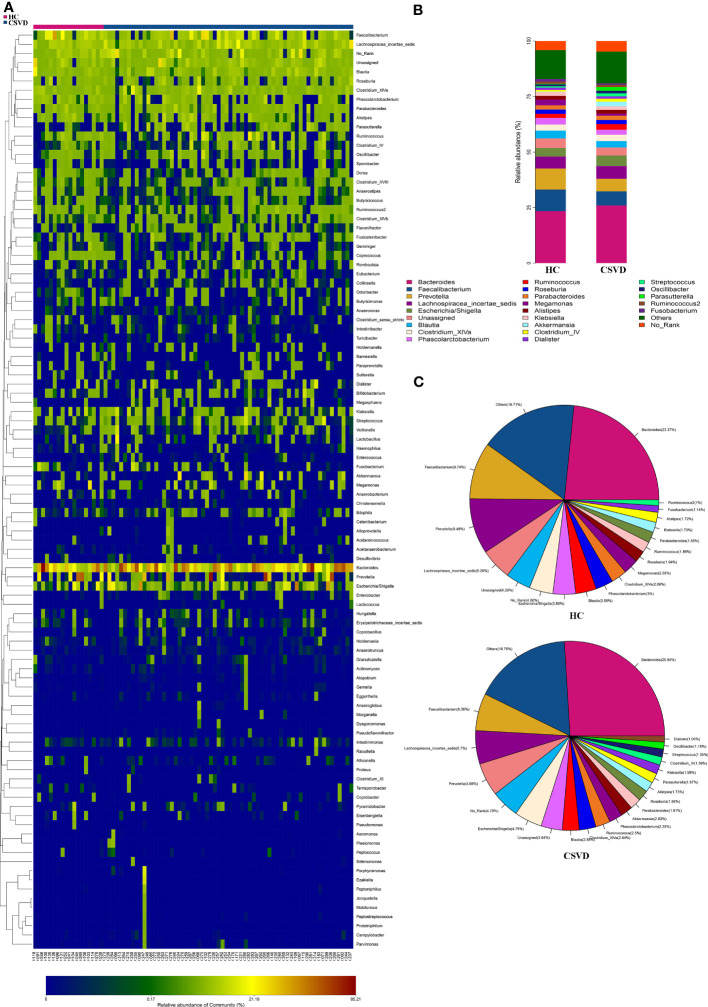
Relative abundances at genus level between CSVD and HC groups. **(A)** Heat-map analysis at genus. Abscissa is the sample and ordinate is the taxa at genus level. The colors in heat-map represent the species abundance, and the gradual change of color from blue to red indicates that the species abundance changed from small to large. **(B)** Bar plots of the relative abundances of two groups at genus level. **(C)** Pieplots of the distribution of the relative abundances of two groups at genus level. CSVD, cerebral small vessel disease; HC, healthy control.

The top four most dominant bacterial genera with the highest relative abundances in both the two groups were consistent, *i.e.*, *Bacteroidetes*, *Faecalibacterium*, *Prevotella*, and *Lachnospiracea_incertae_sedis* (**Figures 1A, B**). Except for *Bacteroidetes* with the same highest abundances between the groups, the second highest abundance was that of *Faecalibacterium* (6.36%), followed by *Lachnospiracea_incertae_sedis* (5.57%) and *Prevotella* (5.68%) in the CSVD group, however, in HCs, *Faecalibacterium* (9.74%) accounted for the second highest relative abundance, followed by *Prevotella* (9.48%) and *Lachnospiracea_incertae_sedis* (5.39%) (**Figure 1C**).

**Figure 2:**
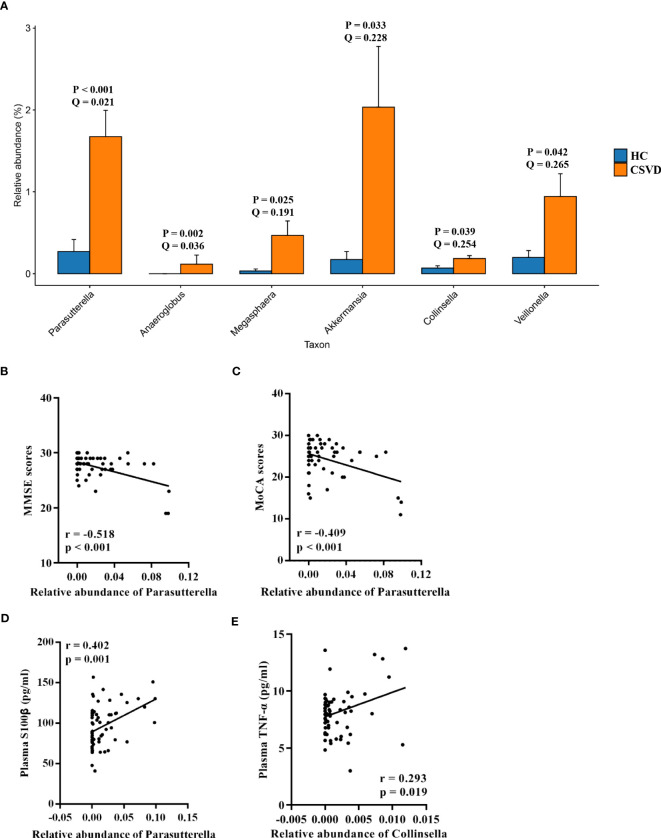
Analysis of taxa with significant differences at genus level. **(A)** Taxa with significant differences at genus level between the CSVD and HC groups. P-value was obtained using Metastats analysis, and Q-value was obtained using FDR correction. **(B, C)** Correlation analysis of MMSE and MoCA scores with taxa with significant differences at genus level in CSVD patients. **(D, E)** Correlation analysis of plasma levels of S100β and TNF-α with taxa with significant differences at genus level in CSVD patients. CSVD, cerebral small vessel disease; HC, healthy control; NIHSS, National Institutes of Health Stroke Scale; MMSE, Mini-mental State Examination; MoCA, Montreal Cognitive Assessment; S100β, S100beta protein; TNF-α, tumor necrosis factor-alpha.

The MetaStats analysis represented six genera with significant differences in their relative abundances between the two groups (**Figure 2A**). *Parasutterella*, *Anaeroglobus*, *Megasphaera*, *Akkermansia*, *Collinsella*, and *Veillonella* significantly increased in the CSVD group as compared to the HC group.

#### LEfSe analysis

Using the LEfSe analysis, there were 17 taxa identified with LDA scores of > 2 and p-value of < 0.05. **Supplementary Figure 3A** showed a cladogram for all the taxonomic levels abundance, and **Supplementary Figure 3B** showed the top 10 taxa with the highest LDA scores in each group. At the genus level, the CSVD group had significantly increased relative abundance of *Parasutterella*, *Enterobacter*, *Terrisporobacter*, *Ezakiella*, and *Anaerostipes* as compared to the HC group, and the HC group had increased relative abundance of *Bacteroides*, *Coprococcus*, *Pseudomonas*, *Dorea*, *Parabacteroides*, and *Phascolarctobacterium* than the CSVD group.

### Correlation analysis of environmental factors in CSVD patients

#### Neuropsychological assessments

As shown in **Figures 2B, C**, the MMSE and MoCA scores were negatively correlated with the relative abundance of *Parasutterella*. However, there was no correlation between the MMSE and MoCA scores and others gut microbiota at genus level levels.

#### Plasma indexes

The relative abundance of *Parasutterella* was positively correlated with plasma levels of S100β (**Figure 2D**). Moreover, a positive correlation was also found between the relative abundance of *Collinsella* and plasma levels of TNF-α (**Figure 2E**).

#### MRI features

**Figure 3:**
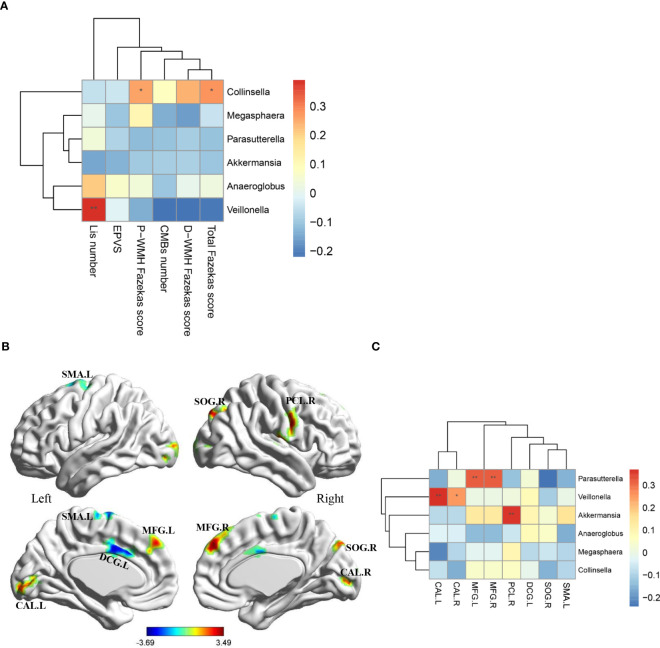
Associations of MRI features with gut microbiota at genus level. **(A)** Heatmap shows the correlation coefficient between structural MRI features and gut microbiota at genus level. **(B)** Eight region with significantly differential ALFF in CSVD patients compared with HCs (p < 0.05, Alphasim multiple comparison correction, voxel number: 70). Covariates were age, gender and years of education. **(C)** Heatmap shows the correlation coefficient between ALFF values in eight brain regions and gut microbiota at genus level. MRI, magnetic resonance imaging; CSVD, cerebral small vessel disease; HC, healthy control; P-WMH, periventricular white matter hyperintensities; D-WMH, deep white matter hyperintensities; EPVS, enlarged perivascular spaces; CMBs, cerebral microbleeds; Lis, lacunar infarcts; ALFF, amplitude of low-frequency fluctuation; SMA, supplementary motor area; DCG, median cingulate and paracingulate gyri; MFG, middle frontal gyrus; CAL, calcarine fissure and surrounding cortex; SOG, superior occipital gyrus; PCL, paracentral lobule. *P-value < 0.05; **P-value < 0.01.

The total Fazekas scores and periventricular WMH Fazekas scores were positively correlated with the relative abundance of *Collinsella*, and the number of Lis was positively correlated with the relative abundance of *Veillonella* (**Figure 3A**).

In addition, compared with HCs, CSVD patients exhibited decreased ALFF values in the left supplementary motor area and left median cingulate and paracingulate gyri, and increased ALFF values in the bilateral middle frontal gyrus, bilateral calcarine fissure and surrounding cortex, right superior occipital gyrus, right paracentral lobule (**Figure 3B**, AlphaSim corrected p < 0.05, number of voxels: 70). Further correlation analyses in CSVD patients revealed that the ALFF values in the bilateral middle frontal gyrus were positively correlated with the relative abundance of *Parasutterella*, the ALFF values in the bilateral calcarine fissure and surrounding cortex were positively correlated with the relative abundance of *Veillonella*, and the ALFF values in the right paracentral lobule were positively correlated with the relative abundance of *Akkermansia* (**Figure 3C**).

### Functional predictions for gut microbiota in the two groups

**Figure 4:**
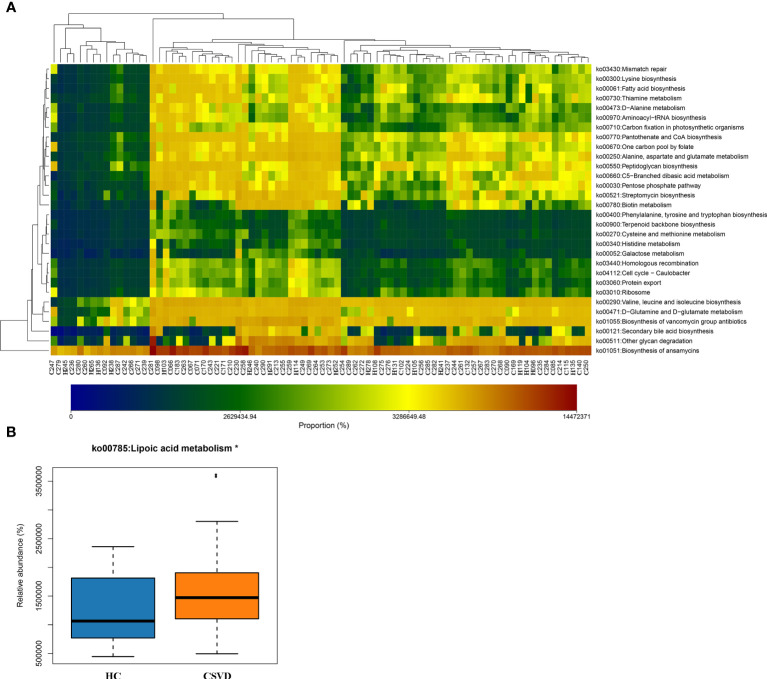
Functional predictions of the gut microbiota. **(A)** Heat-map of functional genes in gut microbiota of all participants, showing top. 30 genes with maximum relative abundances. The Abscissa stands for the samples, and the ordinate is functional genes. The colors represent the abundance of function, and the gradual change in color from light to deep indicates the relative abundance of function from low to high. **(B)** Significant difference in Kyoto Encyclopedia of Genes and Genomes pathways for gut microbiota in CSVD and HC groups. *P-value < 0.05. CSVD, cerebral small vessel disease; HC, healthy control.

Using the PICRUSt2 analysis tool, the species’ functions in the gut microbiota of both the groups were predicted and annotated based on the Kyoto Encyclopedia of Genes and Genomes database. The relative abundances of the functional genes in gut microbiota responsible for the “biosynthesis of ansamycins”, “biosynthesis of vancomycin group antibiotics”, “valine, leucine and isoleucine biosynthesis”, “glycan degradation”, and “D-Glutamine and D-glutamate metabolism” were high (**Figure 4A**). Meanwhile, the differences in the functional prediction between the two groups were analyzed further using Welch’s t-test. The pathway of “lipoic acid metabolism” in the CSVD group was significantly higher than the HC group (**Figure 4B**).

### Diagnostic performance of gut microbiota for CSVD

**Figure 5:**
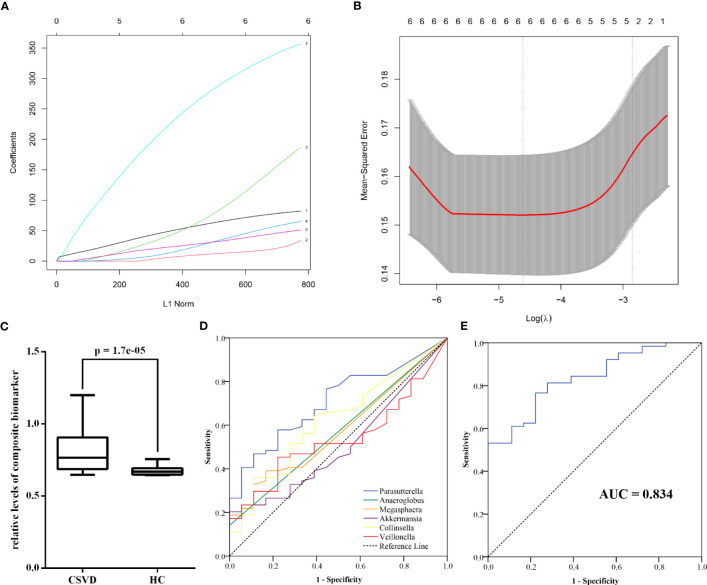
Diagnostic performance of gut microbiota at genus level for CSVD. **(A)** The coefficients of each gut microbiota in the LASSO model (1 = *Parasutterella*, 2 = *Anaeroglobus*, 3 = *Megasphaera*, 4 = *Akkermansia*, 5 = *Collinsella*, 6 = *Veillonella*). **(B)** The mean-squared error of LASSO model. **(C)** The significant difference in the relative levels of the composite biomarker between CSVD patients and HCs. **(D)** ROC curve of six gut microbiota at genus level for identifying CSVD. **(E)** ROC curve of the composite biomarker for identifying CSVD. CSVD, cerebral small vessel disease; HC, healthy control; LASSO, least absolute shrinkage and selection operator; ROC, receiver operating characteristic.

Using the machine learning, a composite biomarker was built based on the gut microbiota at genus level with significant differences between the groups. The composite biomarker of the LASSO model was calculated as follows: *composite biomarker = the relative abundance of Parasutterella × 3.8879931 + the relative abundance of Anaeroglobus × 1.9590061 + the relative abundance of Megasphaera × 3.5400764 + the relative abundance of Akkermansia × 0.6387538 + the relative abundance of Collinsella × 24.9083996 + the relative abundance of Veillonella × 2.3311538 + 0.6438699* (**Figures 5A, B**).

There was the significant difference in the relative levels of the composite biomarker between the CSVD and HC groups (**Figure 5C**). ROC curve indicated that the composite biomarker had an AUC value of 0.834 to identifying CSVD patients from HCs (sensitivity = 76.56%, specificity = 77.78%), which was the higher than other single index of gut microbiota (**Figures 5D, E**; **Supplementary Table 1**).

## Discussion

**Figure 6:**
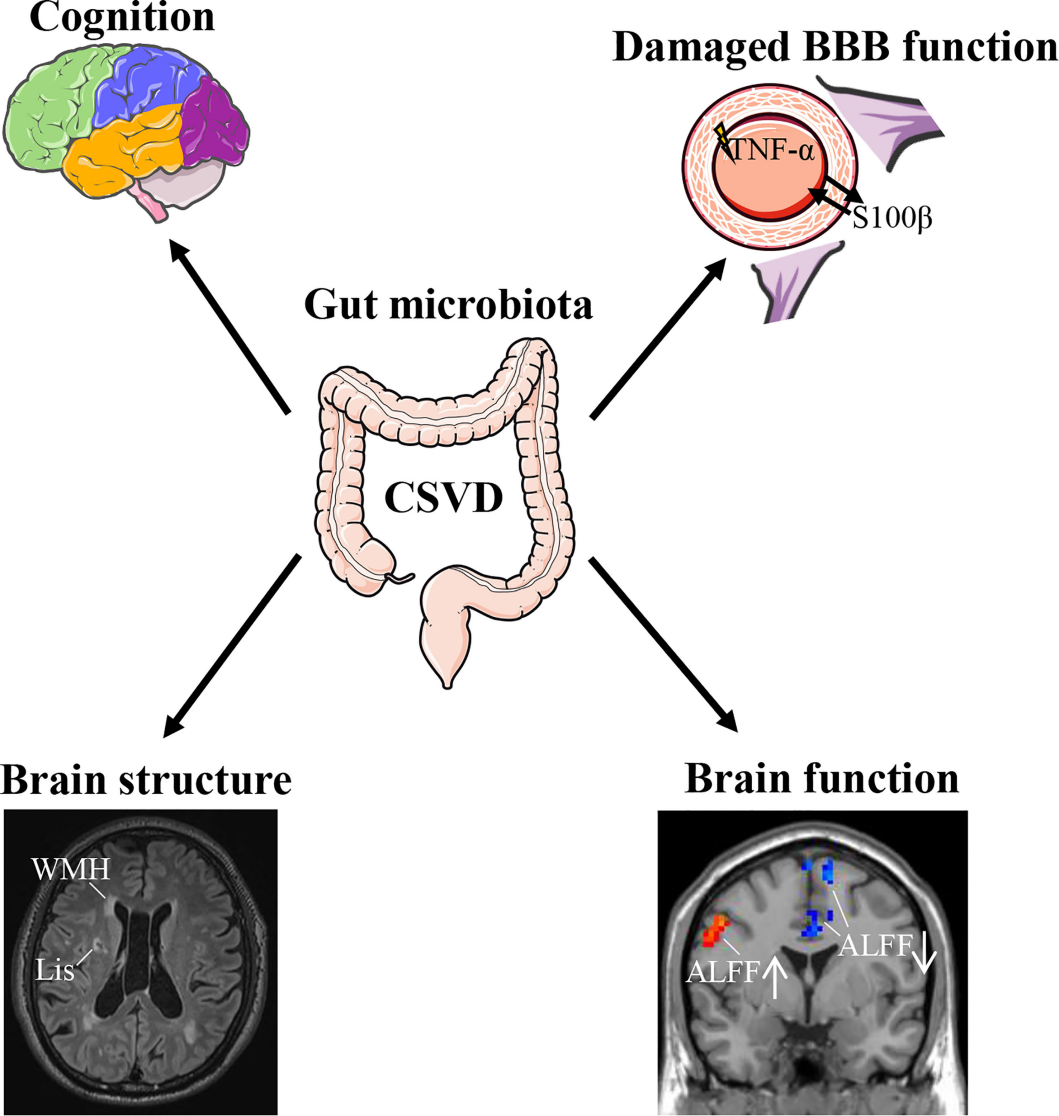
Association of gut microbiota with CSVD. CSVD, cerebral small vessel disease; BBB, blood-brain barrier; WMH, white matter hyperintensities; Lis, lacunar infarcts; ALFF, amplitude of low-frequency fluctuation.

The main findings of the present study were: (1) The CSVD group had significantly lower species diversity and richness in the gut microbiota than the HC group. (2) CSVD patients showed significantly higher genus-level relative abundances of *Parasutterella*, *Anaeroglobus*, *Megasphaera*, *Akkermansia*, *Collinsella*, and *Veillonella* than HCs. (3) Significant associations could be seen between the gut microbiota and cognitive assessments, BBB-/inflammation-related indexes, and structural/functional MRI changes in CSVD patients. (4) Lipoic acid metabolism was involved in CSVD pathogenesis. (5) A composite biomarker of intestinal bacteria revealed the optimal diagnostic power for distinguishing CSVD from HCs. Therefore, the gut microbiota is vital in CSVD pathogenesis (**Figure 6**), with great potential as a clinical biomarker for CSVD diagnosis.

The present study analyzed diversity and potential function in the gut microbiota of CSVD patients. Meanwhile, environmental factors, such as clinical symptoms, peripheral molecular indexes, and brain imaging features, helped comprehensively estimate the association between gut microbiota and CSVD. Furthermore, a machine learning LASSO model was used to build a composite biomarker depending on the gut microbiota at the genus level to improve the CSVD identification accuracy. The rigorous study design and findings strongly support the significance of gut microbiota in CSVD pathological changes. Therefore, we first proposed ideal biomarkers of the gut microbiota for CSVD clinical application.

The *Parasutterella* genus is a core component of the human gut microbiota and is associated with bile acid maintenance and cholesterol metabolism (Ju et al., 2019). Recently, Akash et al. observed that Alzheimer’s disease rat model had an age-related enhancement of *Parasutterella* (Nagarajan et al., 2023). Additionally, a neuroprotective drug, resveratrol, can effectively improve cognitive impairment and increase the abundance of *Parasutterella* in Alzheimer’s disease mice (Yang et al., 2023). Similarly, in our study, CSVD patients had significantly elevated relative abundances compared with HCs. Moreover, significant correlations were observed in CSVD patients between relative abundances of *Parasutterella* and specific clinical features (*e.g.*, MMSE and MoCA scores and the ALFF values of bilateral middle frontal gyrus). Thus, the *Parasutterella*-mediated pathological mechanism may affect cognitive decline (Liang et al., 2022). Furthermore, there was a significantly positive correlation between the relative abundance of *Parasutterella* in CSVD patients and S100β plasma levels, a peripheral BBB function marker (Thelin et al., 2017; Solarz et al., 2021), which suggested that *Parasutterella* may be associated with disrupted BBB integrality in CSVD. Previous studies indicated that high-density lipoproteins protected endothelial function and BBB integrity (Tran-Dinh et al., 2013). However, increased high cholesterol ingestion could exacerbate BBB disruption and affect cognitive function (De Oliveira et al., 2020). Therefore, *Parasutterella* may control the cholesterol metabolism to affect the BBB function, leading to cognitive impairment in CSVD patients.

The relative abundances of *Collinsella* were significantly increased in some psychiatry disorders [*e.g.*, schizophrenia (Li et al., 2020) and autism spectrum disorders (Ding et al., 2020)] compared to HCs in the published studies, indicating that *Collinsella* could affect the normal function of the central nervous system. However, only one study on cerebrovascular disease revealed that combining Puerariae Lobatae Radix and Chuanxiong Rhizoma can enhance intestinal Collinsella disturbances in ischemic stroke (Chen et al., 2019a). The present study observed significantly elevated *Collinsella* levels and a positive correlation with the WMH scores in CSVD patients. This indicated that the relative abundance of *Collinsella* may reflect the CSVD severity. Additionally, *Ruiz-Limón et al.* demonstrated that the increase of the genus *Collinsella* was related to cumulative inflammatory activity (Ruiz-Limón et al., 2022). A significant association was observed in our study between *Collinsella* and plasma TNF-α levels in CSVD patients. Hence, *Collinsella*-mediated inflammation response could have an effect on the CSVD white matter lesions.

A previous study identified more *Akkermansia* in cerebral ischemic stroke patients than HCs (Li et al., 2019). However, another study described contrary results in stroke patients (Chang et al., 2021). In our study, CSVD patients had significantly elevated *Akkermansia* levels compared to HCs. Moreover, *Akkermansia* levels were positively associated with the ALFF values in the right paracentral lobule. Due to lower paracentral lobule activity being negatively associated with the total small vessel disease burden (Xu et al., 2018), *Akkermansia* as a probiotic (Zhang et al., 2019) may protect CSVD.

Furthermore, CSVD patients showed a significantly higher relative abundance of *Veillonella*, *Megasphaera*, and *Veillonella* than HCs. Among them, elevated *Megasphaera* was also observed in stroke patients (Wang et al., 2021), but other gut microbiota were proposed firstly by us in patients with cerebrovascular disease. The present study revealed that significantly increased *Veillonella* exhibited positive correlations with the number of Lis and the ALFF values in the bilateral calcarine fissure and surrounding cortex among CSVD patients. Therefore, *Veillonella* could be associated with CSVD occurrence by affecting brain activity in bilateral calcarine fissure and surrounding cortex. However, the underlying mechanism of these gut microbiota in CSVD pathology remains unknown.

A combined biomarker using the LASSO model showed better diagnostic performance in identifying CSVD than the single gut microbiota biomarker. Thus, using machine learning contributes to elevating the differential power for CSVD.

The present study had some limitations. (1) The number of HCs was less than the CSVD group. A sample size calculator was used (https://sample-size.net/), which indicated the acceptability of the current sample size. The sample size will be increased in subsequent studies to verify the obtained results. (2) Since CSVD patients possess various clinical symptoms, the different clinical subtypes of CSVD may possess distinct performances of gut microbiota. We did not analyze the differences in gut microbiota among CSVD patients with cognitive impairment, depression, or gait disturbances. (3) The independent present composite biomarker verification of gut microbiota is absent in the present study. (4) Animal study is absent in the present study. We will conduct the animal study to determine the association between the gut microbiota and CSVD pathological mechanisms in the following study. Therefore, a multi-center study should be conducted to validate the current findings.

## Conclusion

The present study demonstrated the composition of gut microbiota composition in patients with CSVD and found several microbes correlated with cognitive decline, BBB integrality, and brain MRI changes, which might expound the underlying pathogenesis of CSVD. Meanwhile, a composite biomarker of gut microbiota using LASSO model will contribute to identifying CSVD conveniently and accurately.

## Data availability statement

The datasets presented in this study can be found in online repositories. The names of the repository/repositories and accession number(s) can be found below: The sequencing data can be obtained from GEO database (SRA accession number: PRJNA985039; https://www.ncbi.nlm.nih.gov/bioproject/PRJNA985039).

## Ethics statement

The studies involving human participants were reviewed and approved by the Ethics Committee of the Affiliated Wuxi People’s Hospital of Nanjing Medical University (approval number: KY2112). The patients/participants provided their written informed consent to participate in this study. Written informed consent was obtained from the individual(s) for the publication of any potentially identifiable images or data included in this article.

## Author contributions

YS, XF, and FW designed the study. YS and EZ draft the manuscript. HM, QG, SiZ, and LM collected the image data. LL, JD, YL, GX, and YY recruited the participants, completed the neuropsychological assessments, and collected the blood samples. LL and SoZ analyzed the data. JS contributed to the discussion. XF and FW contributed to the discussion and revised the manuscript. All authors contributed to the article and approved the submitted version.
