# Supplementary material for: Alteration and clinical potential in gut microbiota in patients with cerebral small vessel disease
Source: Front Cell Infect Microbiol. 2023 Jul 11;13:1231541. doi: 10.3389/fcimb.2023.1231541 (PMC10366612; doi:10.3389/fcimb.2023.1231541)
Supplement: Supplementary file 1 [file DataSheet_1.docx]

#### Image acquisition and data preprocessing

Data acquisition and preprocessing for each dataset have been described in detail in our previous studies ([Shi et al., 2021a](#_ENREF_5); [Shi et al., 2021b](#_ENREF_6); [Shi et al., 2022](#_ENREF_4)). A 3.0T MR scanner (MAGNETOM Prisma, Siemens Healthcare, Germany) is used in the present study. The data of high-resolution T1-weighted images (T1WI) and resting-state functional MRI (rs-fMRI) was collected. None of subjects had excessive motion artifacts (≥ 2 mm translational or ≥ 2° rotational movements) or incomplete image coverage.

The scanning parameters were as follows: Protocol name = EPI; Repetition time = 1500 ms; Echo time = 31 ms; Flip angle = 70°; Field of view = 211 mm × 211 mm; Matrix = 88 × 88; Thickness (gap) = 2.4 mm (0); Slice number = 60.

#### Rs-fMRI data analysis

Rs-fMRI data were preprocessed using SPM8 and the Data Processing Assistant for Resting-State fMRI (DPARSF, <http://www.restfmri.net/forum/dparsf>) ([Chao-Gan and Yu-Feng, 2010](#_ENREF_2)). The initial 10 functional volumes were discarded for scanner stabilization and participant adaption. The remaining images were corrected for timing differences and motion effects. The individual structural images (T1WI) were co-registered to mean functional images after motion correction using linear transformation. Motion corrected functional volumes were spatially normalized to the Montreal Neurological Institute space using DARTEL toolbox ([Ashburner, 2007](#_ENREF_1)) and resampled to a voxel size of 3 mm × 3 mm × 3 mm. To further reduce the effects of confounding factors, Friston 24 motion parameters ([Friston et al., 1996](#_ENREF_3)), white matter signal, and cerebrospinal fluid signal were removed from the data via linear regression. Then, smoothing with a 6-mm full-width at half-maximum kernel and linear detrending were performed.

#### Amplitude of low-frequency fluctuation (ALFF) analyses

DPARSF software was used for the present analyses ([Chao-Gan and Yu-Feng, 2010](#_ENREF_2)). Briefly, for each voxel, the time series was transformed to the frequency domain using Fast Fourier Transform and the power spectrum was acquired. Next, the square root was computed at each frequency of the power spectrum and average square root was obtained across 0.01 - 0.08 Hz at each voxel. The averaged square root was used as the value of ALFF. To reduce the global influence of variability across patients, the ALFF of each voxel was divided by the global mean ALFF value for each subject using a pre-defined cerebral gray matter mask.

#### Statistical analysis of MRI data

REST software was used for the analysis of MRI data across groups and group differences in whole-brain ALFF values (using the AAL_90 template). Two-sample t-test was used for assess the between-group differences in ALFF values across CSVD and HC groups and a voxel-wise one-way analysis of covariance (ANCOVA) was performed to examine CSVD-related alterations in ALFF maps, with age, gender and years of education as covariates. The results were considered significant differences at a corrected p < 0.05 using Alphasim multiple comparison correction and cluster size > 70 voxels. Mean ALFF values of clusters exhibiting significant differences between groups were extracted for HCs, which was exhibited using the BrainNet Viewer software (<http://www.nitrc.org/projects/bnv/>).

#### 16S rRNA gene sequencing

The DNA from fecal samples (500 mg for each sample) was extracted using FastDNA Spin Kit For Soil (MP Biomedicals, Santa Ana, CA) following the manufacturer’s guidelines. The integrity and quality of genomic DNA were determined by agarose gel electrophoresis, and NanoDrop 2000 (10x Genomics, USA) and Invitrogen Qubit 3.0 Spectrophotometer (Thermo Fisher Scientific, USA), respectively. The V3-V4 hyper-variable regions of the 16S rRNA gene were amplified with the primers 341F (5’-CCTACGGGNGGCWGCAG-3’) and 805R (5’-GACTACHVGGGTATCTAATCC-3’) using a high-fidelity polymerase chain reaction (PCR). The DNA mix of the standard bacterial genomes as a positive control was used for 16S rRNA gene sequencing. The uniqueness and specificity of the above-mentioned amplified products were examined with agarose gel electrophoresis. The PCR products were purified using Agencourt AMPure XPPCR Purification Beads (Beckman Coulter, USA). The concentration of the index labeled sample gene library was diluted 5-10 times according to the preliminary quantitative result shown by agarose gel using Qubit. The length of the inserted fragment was measured using Agilent 2100 Bioanalyzer (Agilent Technologies, USA). Illumina NovaSeq 6000 (Illumina, USA) sequencing platform was utilized to sequence the library.

#### Data processing and analysis of sequencing

The raw sequence reads were analyzed with Quantitative Insights Into Microbial Ecology (QIIME 2). The Cutadapt plugin was utilized to tailor the adaptor and primer sequences. The quality control and identification of amplicon sequence variants (ASVs) were conducted using Divisive Amplicon Denoising Algoruthm 2 (DADA2). The taxonomic assignments of ASV representative sequences were conducted according to a confidence threshold of 0.8 using a pre-trained Naive Bayes classifier, which was trained on the Ribosomal Database Project (RDP) classifier (version 11.5).

## Supplementary Table 1. The ROC curve analysis of gut microbiota for identifying CSVD patients from HCs.

|  | Area under the curve | Sensitivity (%) | Specificity (%) |
| --- | --- | --- | --- |
| Composite biomarker | 0.834 | 76.56 | 77.78 |
| Parasutterella | 0.707 | 46.88 | 88.89 |
| Anaeroglobus | 0.570 | 14.06 | 100.00 |
| Megasphaera | 0.576 | 39.06 | 83.33 |
| Akkermansia | 0.510 | 20.31 | 100.00 |
| Collinsella | 0.622 | 64.06 | 61.11 |
| Veillonella | 0.536 | 45.31 | 77.78 |


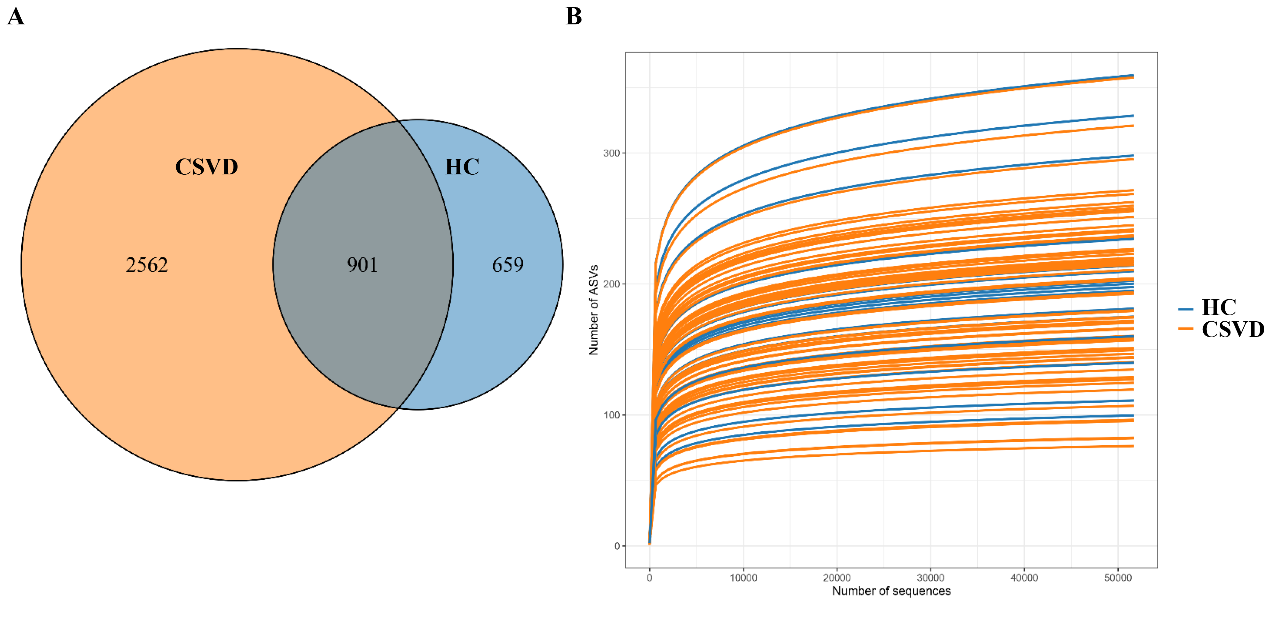


**Supplementary Figure 1. Sequencing data of gut microbiota in 82 participants**

(A) Venn of the distribution of gut microbiota in the two groups. (B) All the samples’ curves in the rarefaction curves.


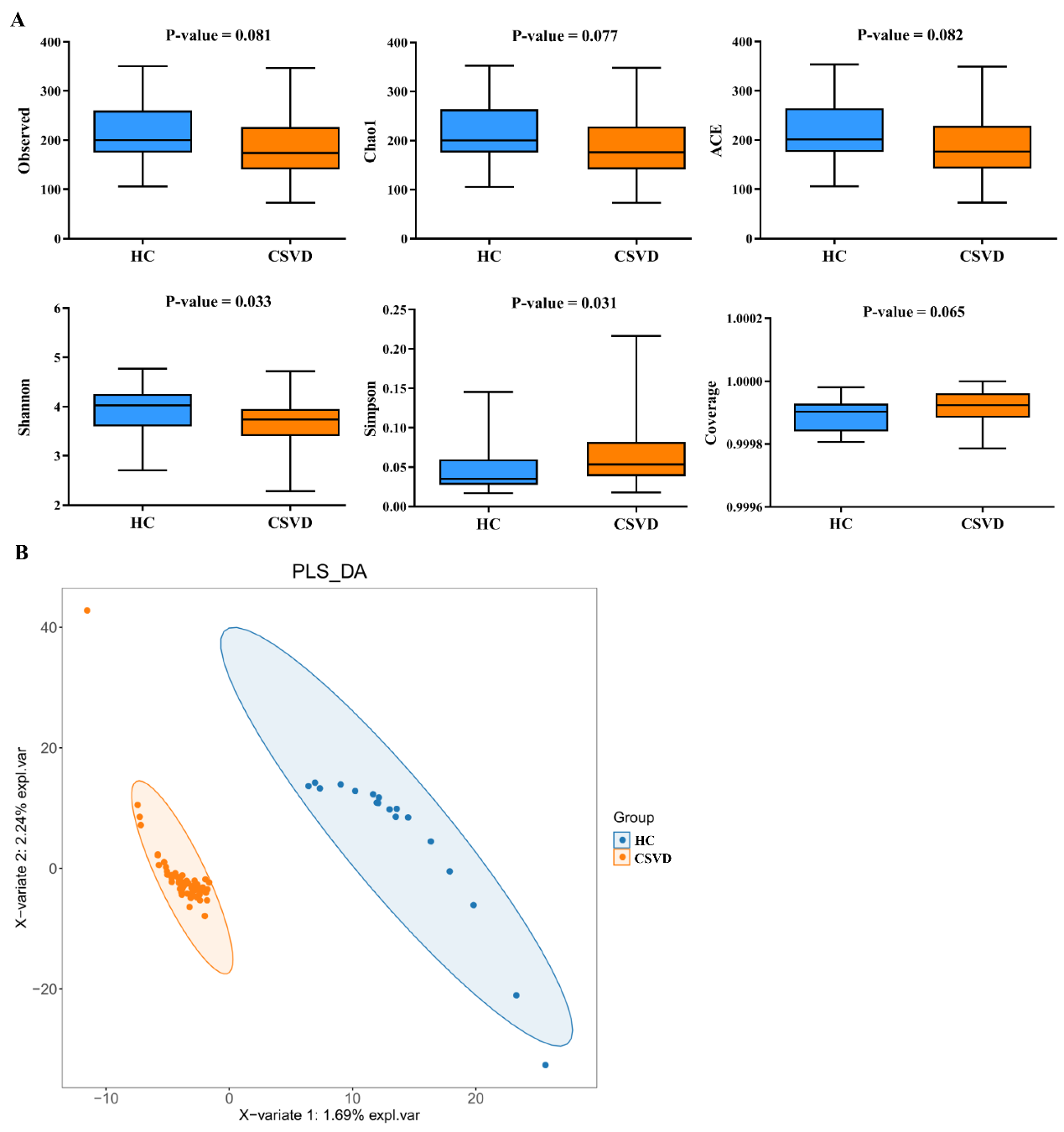


**Supplementary Figure 2. Alpha-diversity and Beta-diversity indices for the species in the gut microbiota of CSVD and HC groups.**

(A) α-diversity indices.

(B) β-diversity indices using PLS-DA analysis: closer the distance between two points, more similar the composition of two samples. The scale stands for the relative distance without practical significance. The HC and CSVD subjects are colored in blue and orange, respectively.


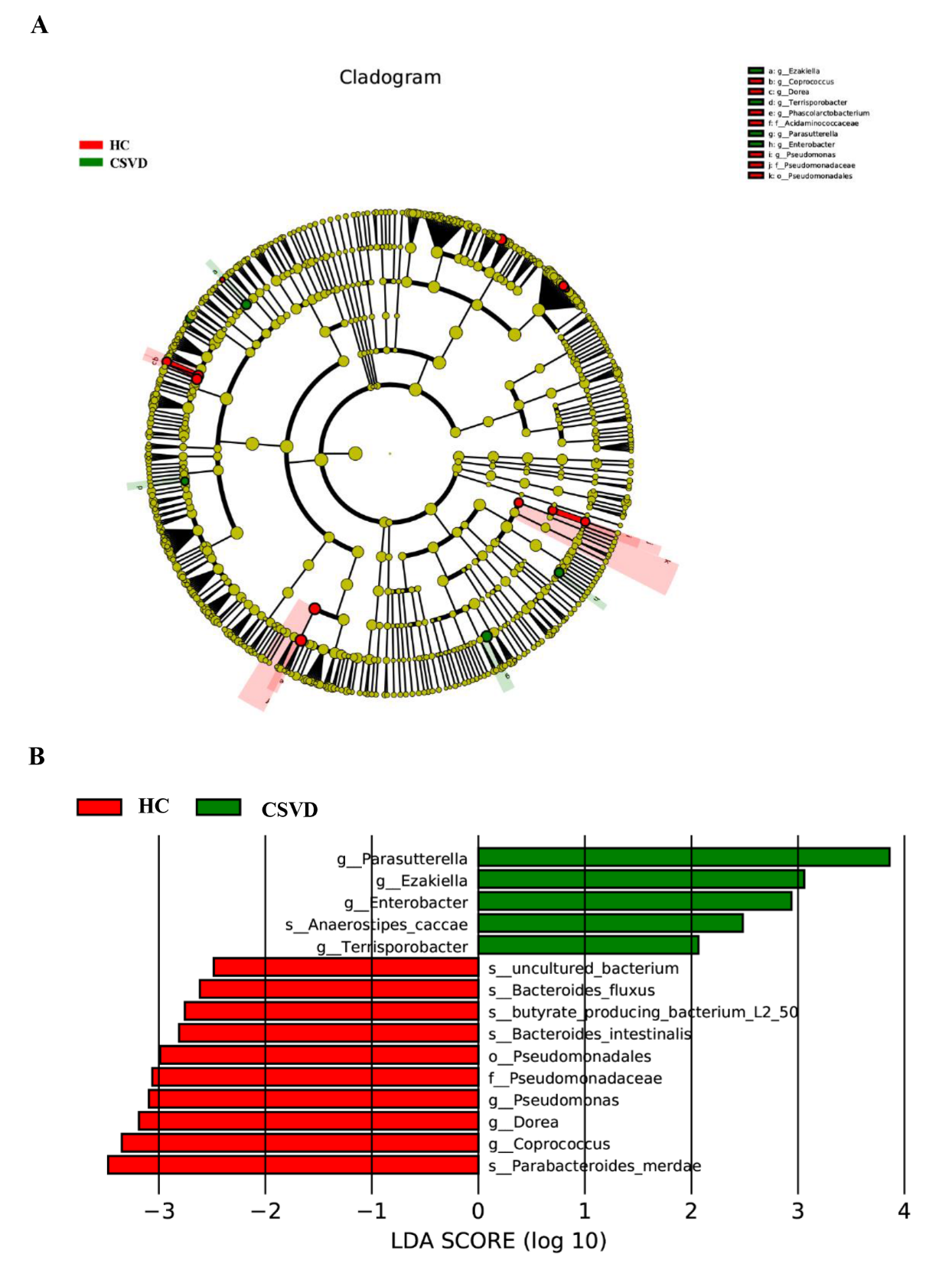


**Supplementary Figure 3. Taxa with different abundances according to LEfSe analysis in the CSVD and HC groups.**

(A) Cladogram generated by LEfSe. Statistically significant differences (LDA scores > 2) in relative abundance of taxa with the minimum P-value between the CSVD and HC groups, P < 0.05. Red and green nodes indicate enriched taxa in the CSVD and HCn groups, respectively. The diameter of each node shows the relative abundance of each taxon and is proportional to the observed effect size. (B) Histogram of the LDA scores (>2) computed for the top 10 taxa with minimum P-value. Red and green bars indicate taxa were enrichment in the CSVD and HC groups, respectively.

**Reference**

Ashburner J (2007). A fast diffeomorphic image registration algorithm. Neuroimage, 38(1): 95-113

Chao-Gan Y, Yu-Feng Z (2010). DPARSF: A MATLAB Toolbox for "Pipeline" Data Analysis of Resting-State fMRI. Frontiers in systems neuroscience, 4: 13

Friston K J, Williams S, Howard R, Frackowiak R S, Turner R (1996). Movement-related effects in fMRI time-series. Magnetic Resonance in Medicine, 35(3): 346-355

Shi Y, Mao H, Gao Q, Xi G, Zeng S, Ma L, Zhang X, Li L, Wang Z, Ji W, He P, You Y, Chen K, Shao J, Mao X, Fang X, Wang F (2022). Potential of brain age in identifying early cognitive impairment in subcortical small-vessel disease patients. Frontiers in Aging Neuroscience, 14: 973054

Shi Y, Song R, Wang Z, Zhang H, Zhu J, Yue Y, Zhao Y, Zhang Z (2021a). Potential clinical value of circular RNAs as peripheral biomarkers for the diagnosis and treatment of major depressive disorder. EBioMedicine, 66: 103337

Shi Y, Zhang L, He C, Yin Y, Song R, Chen S, Fan D, Zhou D, Yuan Y, Xie C, Zhang Z (2021b). Sleep disturbance-related neuroimaging features as potential biomarkers for the diagnosis of major depressive disorder: A multicenter study based on machine learning. J Affect Disord, 295: 148-155
